# Supplementary material for: Chronic Lymphocytic Leukemia (CLL) with Borderline Immunoglobulin Heavy Chain Mutational Status, a Rare Subgroup of CLL with Variable Disease Course
Source: Cancers (Basel). 2024 Mar 8;16(6):1095. doi: 10.3390/cancers16061095 (PMC10969134; doi:10.3390/cancers16061095)
Supplement: Supplementary file 1 [file cancers-16-01095-s001.zip › cancers-2865071-supplementary.pdf]

Supplementary Table S1: Univariate Cox regression analysis for TTFT

Variable	Hazard Ratio	95% CI	P values
Sex (M vs F)	1.22	0.98 – 1.52	0.072
Age	1.02	1.01 – 1.03	**<0.001**
RAI stage (0-I vs II-IV)	0.43	0.35 – 0.54	**<0.001**
BINET stage (A vs B-C)	0.35	0.29 – 0.44	**<0.001**
IGHV mutational status			
M-IGHV	ref	-	**-**
BL-IGHV	3.76	2.85 – 5.93	**<0.001**
U-IGHV	3.96	3.17 – 5.02	**<0.001**
del(11q)	3.48	2.58 – 4.70	**<0.001**
del(13q)	0.67	0.53 – 0.84	**<0.001**
+12	1.28	0.94 – 1.75	0.116
del(17p)	2.88	2.03 – 4.09	**<0.001**
TP53	2.65	1.78 – 3.96	**<0.001**
NOTCH1	1.21	0.78 – 1.89	0.399
Karyotype			
Normal	ref	-	-
<2 abnormalities	1.80	1.24 – 2.59	**0.002**
CK	1.83	1.11 – 3.00	**0.018**
High-CK	2.96	1.82 – 4.81	**<0.001**

Supplementary Table S2: Multivariate Cox regression analysis for TTFT

Variable	Hazard Ratio	95% CI	P values
Age	1.04	1.02 – 1.06	**<0.001**
RAI stage (0-I vs II-IV)	0.82	0.36 – 1.84	0.639
BINET stage (A vs B-C)	0.91	0.38 – 2.16	0.830
IGHV mutational status			
M-IGHV	ref	-	**-**
BL-IGHV	2.60	1.21 – 5.58	**0.013**
U-IGHV	1.64	1.03 – 2.60	**0.037**
del(11q)	1.59	0.96 – 2.65	0.072
del(13q)	0.61	0.40 – 0.94	**0.023**
del(17p)	0.60	0.25 – 1.41	0.239
TP53	2.14	1.21 – 4.07	**0.021**
NOTCH1	1.31	0.74 – 2.31	0.350
Karyotype			
Normal	ref	-	-
<2 abnormalities	1.26	0.75 – 2.11	0.374
CK	1.70	0.90 – 3.20	0.102
High-CK	2.31	1.12 – 4.74	**0.023**

Supplementary Table S3: Univariate Cox regression analysis for OS

Variable	Hazard Ratio	95% CI	P values
Sex (M vs F)	1.66	1.24 – 2.21	**<0.001**
Age	1.08	1.06 – 1.09	**<0.001**
RAI stage (0-I vs II-IV)	0.56	0.42 – 0.73	**<0.001**
BINET stage (A vs B-C)	0.46	0.35 – 0.60	**<0.001**
IGHV mutational status			
M-IGHV	ref	-	**-**
BL-IGHV	0.97	0.40 – 2.40	0.956
U-IGHV	2.86	2.17 – 3.76	**<0.001**
del(11q)	3.02	2.03 – 4.48	**<0.001**
del(13q)	0.72	0.52 – 0.99	**0.049**
+12	1.15	0.74 – 1.78	0.532
del(17p)	1.87	1.15 – 3.03	**0.011**
TP53	1.30	0.66 – 2.58	0.449
NOTCH1	1.95	0.93 – 4.07	0.076
Karyotype			
Normal	ref	-	-
<2 abnormalities	1.16	0.61 – 2.24	0.651
CK	1.63	0.75 – 3.56	0.217
High-CK	2.90	1.43 – 5.90	**0.003**

Supplementary Table S4: Multivariate Cox regression analysis for OS

Variable	Hazard Ratio	95% CI	P values
Sex (M vs F)	2.42	1.09 – 5.41	**0.031**
Age	1.12	1.07 – 1.17	**<0.001**
RAI stage (0-I vs II-IV)	0.93	0.28 – 3.11	0.905
BINET stage (A vs B-C)	0.52	0.15 – 1.77	0.292
IGHV mutational status			
M-IGHV	ref	-	**-**
BL-IGHV	0.64	0.72 – 5.63	0.686
U-IGHV	1.53	0.60 – 3.89	0.370
del(11q)	1.69	0.66 – 4.32	0.275
del(13q)	0.99	0.46 – 2.15	0.996
del(17p)	0.61	0.13 – 2.90	0.535
TP53	1.78	0.59 – 5.43	0.308
NOTCH1	2.61	0.95 – 7.17	0.063
Karyotype			
Normal	ref	-	-
<2 abnormalities	0.80	0.28 – 2.31	0.682
CK	0.93	0.25 – 3.48	0.916
High-CK	2.00	0.57 – 6.97	0.276

Supplementary Table S5: Frequency of IGHV gene families by IGHV mutational status, only IGHV with a frequency of ≥1% in any group are shown.

**IGHV gene family n (%)**	**M-IGHV**	**BL-IGHV**	**U-IGHV**	**P values***
IGHV 1-2	13 (3.7)	0	20 (7.3)	**0.049**
IGHV 1-3	6 (1.7)	0	4 (1.5)	1
IGHV 1-8	6 (1.7)	0	3 (1.1)	1
IGHV 2-5	10 (2.9)	2 (6.7)	1 (0.4)	**0.01**
IGHV 1-69	9 (2.6)	1 (3.3)	54 (19.9)	**< 0.01**
IGHV 3-11	6 (1.7)	0	9 (3.3)	0.39
IGHV 3-15	12 (3.4)	1 (3.3)	4 (1.5)	0.26
IGHV 3-21	13 (3.7)	7 (23.3)	10 (3.7)	**< 0.01**
IGHV 3-23	38 (10.9)	5 (16.7)	10 (3.7)	**< 0.01**
IGHV 3-30	28 (8.0)	1 (3.3)	12 (4.4)	0.27
IGHV 3-33	10 (2.9)	0	13 (4.8)	0.35
IGHV 3-48	12 (3.4)	2 (6.6)	13 (4.8)	0.39
IGHV 3-49	3 (0.9)	0	3 (1.1)	1
IGHV 3-53	7 (2.0)	0	3 (1.1)	0.53
IGHV 3-7	31 (8.9)	2 (6.6)	5 (1.8)	**< 0.01**
IGHV 3-9	2 (0.6)	1 (3.3)	6 (2.2)	0.11
IGHV 3-72	7 (2.0)	0	0	0.06
IGHV 3-73	1 (0.3)	0	4 (1.5)	0.35
IGHV 3-74	11 (3.1)	4 (13.3)	3 (1.1)	**< 0.01**
IGHV 4-31	8 (2.3)	0	3 (1.1)	0.62
IGHV 4-34	33 (9.4)	1 (3.3)	11 (4.0)	**< 0.01**
IGHV 4-39	9 (2.6)	0	7 (2.6)	1
IGHV 4-4	8 (2.3)	0	4 (1.5)	0.75
IGHV 4-59	19 (5.4)	1 (3.3)	10 (3.7)	0.59
IGHV 4-61	9 (2.6)	0	3 (1.1)	0.88
IGHV 5-51	4 (1.1)	0	14 (5.1)	**< 0.01**
Not available	18 (5.1)	0	18 (6.6)	0.34

* Overall p-values between the three groups, relevant pair-wise p-values reported in text; p-values below the significance level are indicated in bold.

Supplementary Table S6: BCR stereotype subsets frequency by IGHV mutational status

**Subset n (%)**	**M-IGHV**	**BL-IGHV**	**U-IGHV**	**P values***
# 1	0	0	6 (2.2)	**0.02**
# 2	9 (2.6)	3 (10)	2 (0.7)	**< 0.01**
# 5	0	0	1 (0.4)	0.46
# 6	0	0	1 (0.4)	0.46
# 8	0	0	1 (0.4)	0.46
# 12	0	0	1 (0.4)	0.46
# 28A	0	0	1 (0.4)	0.46
# 31	0	0	1 (0.4)	0.46
# 64B	1 (0.3)	0	0	**1**
# 77	4 (1.1)	0	0	0.28
# 99	1 (0.3)	0	2 (0.7)	0.64
# 202	0	0	3 (1.1)	0.20

* Overall p-values between the three groups, relevant pair-wise p-values reported in text; p-values below the significance level are indicated in bold.
